# Is surgical axillary staging necessary in women with T1 breast cancer who are treated with breast-conserving therapy?

**DOI:** 10.1186/s40880-019-0371-y

**Published:** 2019-05-08

**Authors:** Jin Wang, Hailin Tang, Xing Li, Cailu Song, Zhenchong Xiong, Xi Wang, Xiaoming Xie, Jun Tang

**Affiliations:** Department of Breast Oncology, State Key Laboratory of Oncology in South China, Collaborative Innovation Center for Cancer Medicine, Sun Yat-sen University Cancer Center, No. 651 Dongfeng East Road, Yuexiu District, Guangzhou, 510060 Guangdong P.R. China

**Keywords:** Surgical axillary staging, T1 breast cancer, Breast-conserving therapy, Surveillance, Epidemiology, and End Results

## Abstract

**Background:**

In the post-Z0011 trial era, the need to perform surgical axillary staging for early-stage breast cancer patients, who are treated with breast-conserving therapy (BCT), is being questioned. We conducted a retrospective cohort study using the Surveillance, Epidemiology, and End Results (SEER) database to evaluate the safety of waiving surgical axillary staging in patients with T1 breast cancer treated with BCT.

**Methods:**

A total of 166,615 eligible patients diagnosed between 2000 and 2012 were divided into staging (sentinel lymph node biopsy or axillary lymph node dissection) and non-staging (no lymph node examined or only needle aspiration biopsy of lymph nodes) groups. Propensity score matching (PSM) was performed to balance disparities between the two groups. Multivariate analysis with the Cox proportional hazards model was used to assess factors related to breast cancer-specific survival (BCSS).

**Results:**

Although the tumor size at time of presentation was decreasing over years, the rate of surgical axillary staging increased from 93.3% to 96.9%. The 5-year BCSS rates of the whole cohort (before PSM) and matched cohort (after PSM) were 98.0% and 97.5%. Within the matched cohort, the BCSS was significantly longer in the staging group than in the non-staging group (*P* < 0.001). However, surgical axillary staging did not benefit patients who were 50–79 years old, had tumor size < 1 cm, histological grade I disease, or favorable histological types (tubular/mucinous/papillary) in stratified analyses (*P* > 0.05). Race, marital status, hormone receptors, and chemotherapy were not associated with the favorable impact of surgical axillary staging on BCSS (*P* > 0.05).

**Conclusion:**

Although surgical axillary staging remains important for T1 breast cancer patients treated with BCT, it might be unnecessary for patients with old age, small tumor, grade I disease, or favorable histological types.

**Electronic supplementary material:**

The online version of this article (10.1186/s40880-019-0371-y) contains supplementary material, which is available to authorized users.

## Background

With improvements in breast cancer screening, increasing numbers of patients are being diagnosed at an early stage with reduced axillary lymph node involvement [1]. As such, surgical treatment of primary breast cancer has de-escalated over the last decades, with breast-conserving surgery (BCS) and sentinel lymph node biopsy (SLNB) being increasingly performed over mastectomy and axillary lymph node dissection (ALND) [2]. Currently, ALND is performed only if the result of SLNB is positive [3]. The International Breast Cancer Study Group (IBSCG) 23-01 trial demonstrated no local control or survival advantages associated with ALND, even in women with micrometastatic SLNs [4]. Furthermore, both the American College of Surgeons Oncology Group (ACOSOG) Z0011 trial and the European Organization for Research and Treatment of Cancer (EORTC) AMAROS trial indicated that ALND could be safely omitted in most patients with 1–2 metastatic SLNs [5, 6].

Although SLNB is highly reproducible, accurate, and associated with reduced morbidity, it is not a risk-free procedure [7]. A 4%–14% rate of complications, such as allergic reactions, hematoma, lymphedema, paresthesia, chronic pain, and immobility, still occurs after SLNB [8–11]. Additionally, the false negative rate of axillary lymph node status predicted by SLNB is 5%–10%, despite the axillary recurrence rate being only 0.3% [3, 12–14]. Taken together, the value of surgical axillary staging for early-stage breast cancer treated with breast-conserving therapy (BCT) remains controversial in the current era of personalized medicine.

The shift in the size of breast tumors is believed to be associated with the increasing use of screening mammography [15]. Until 1999, the average tumor size at initial presentation (stage I–III) has decreased by 10% every 5 years for two decades [16]. However, the rates of T1 tumors (≤ 2 cm) remained relatively unchanged for the past 15 years, and the average tumor size was approximately 1.8 cm [17]. Therefore, using the Surveillance, Epidemiology, and End Results (SEER) database, we aimed to investigate the safety of waiving surgical axillary staging in patients with T1 breast cancer who are treated with BCT.

## Patients and methods

### Data source

We performed a retrospective cohort study using the SEER custom database (http://www.seer.cancer.gov) (with additional datasets of treatment information, released in April 2017) from the US National Cancer Institute. The SEER database currently includes incidence and survival data collected from 18 population-based cancer registries, which covers approximately 28% of the US population [18].

Female patients diagnosed with breast cancer between January 1, 2000 and December 31, 2012, who met the following criteria, were deemed eligible: (1) had T1 breast cancer; (2) had breast cancer as the primary cancer; and (3) were older than 18 years. Since the recurrence rate and breast cancer-related death rate are unacceptably high when patients are treated with BCS without radiotherapy [19], meeting the Z0011 eligibility criteria, patients who underwent BCS alone were not included in the present study. Exclusion criteria were as follows: (1) the patient had received neoadjuvant therapy (identified using the codes “CS Tumor Size/Ext Eval” and “CS Reg Node Eval” from the Collaborative Stage Data Set); (2) the patient had other simultaneous primary malignant tumor; (3) the patient did not receive cancer-directed surgery at primary site; (4) the type of surgery was unknown; (5) the number of lymph nodes examined was unknown; (6) the patient had metastatic lymph nodes on needle aspiration biopsy, but did not receive further axillary treatment; (7) the patient was diagnosed at autopsy; (8) the follow-up data were unavailable.

### Main variables and endpoints

Using the SEER*STAT software version 8.3.4 (Information Management Services, Inc., Calverton, MD, USA), we extracted demographic (year of diagnosis, age, race and origin, and marital status), clinicopathologic (TNM stage classified according to the 6th edition of the American Joint Committee on Cancer staging system, grade, histological type, estrogen receptor, and progesterone receptor), and therapeutic information (surgery of primary site, radiotherapy, chemotherapy, number of regional nodes examined, and number of metastatic regional nodes), along with survival data (cause-specific death classification and survival duration).

According to the “surgery codes of breast C50.0-C50.9”, breast surgeries were classified into BCS and mastectomy. The “number of regional lymph nodes examined” codes (SEER Program Coding and Staging Manual 2016) were used to divide patients into staging and non-staging groups. In particular, we categorized the tumor histology into four types, namely ductal, lobular, favorable (tubular/mucinous/papillary), and others, according to ICD-O-3 codes. The primary outcome was breast cancer-specific survival (BCSS) [20], which was measured from the date of diagnosis to the date for which “cause-specific death” data were available.

### Statistical analysis

Patient characteristics were compared between the staging and non-staging groups using Pearson’s Chi-square test for categorical variables. Temporal trends were assessed using the Cochran–Armitage test. Propensity score matching (PSM) was performed to balance disparities between the two groups. Propensity score for the status of surgical axillary staging was calculated for each patient using multivariate logistic regression, considering all imbalanced factors. We performed a 5-to-1 digit greedy match algorithm at a 1:1 ratio to estimate the propensity score without replacement [21]. Considering that some information (such as endocrine therapy) was not available in the SEER database, we also conducted sensitivity analysis to examine the impact of various levels of hidden bias on the interpretation of treatment effect [22].

The Kaplan–Meier method was used to plot BCSS curves, and log-rank test was performed for comparison of survival. Significant prognostic factors in the univariate analysis were included in the Cox proportional hazards regression model for multivariate analyses. Hazard ratios (HR) from the final models are presented with 95% confidence intervals (CI). Statistical analysis was performed using the SAS version 9.4 software (SAS Institute, Cary, NC, USA) and R version 3.2.0 software (R Foundation for Statistical Computing, Vienna, Austria). Statistical significance was defined as a two-sided *P* value < 0.05.

## Results

### Patient characteristics

**Fig. 1:**
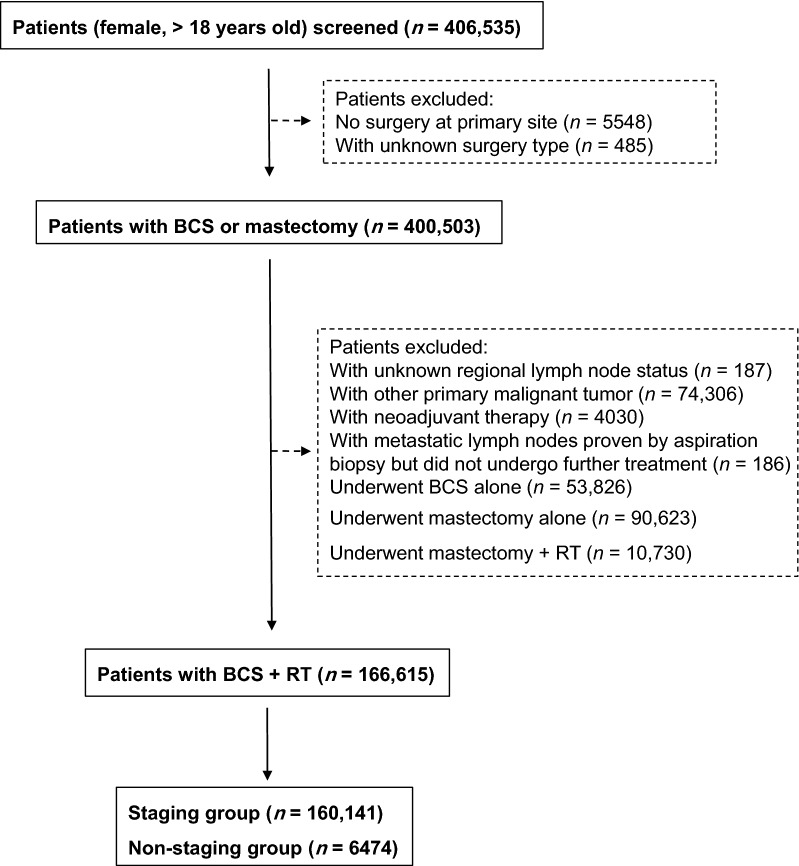
Flow diagram of identifying eligible patients with pT1 breast carcinoma diagnosed between 2000 and 2012 from the Surveillance, Epidemiology, and End Results (SEER) database (November 2016 Submission). *BCS*: breast-conserving surgery, *RT* radiotherapy

**Fig. 2:**
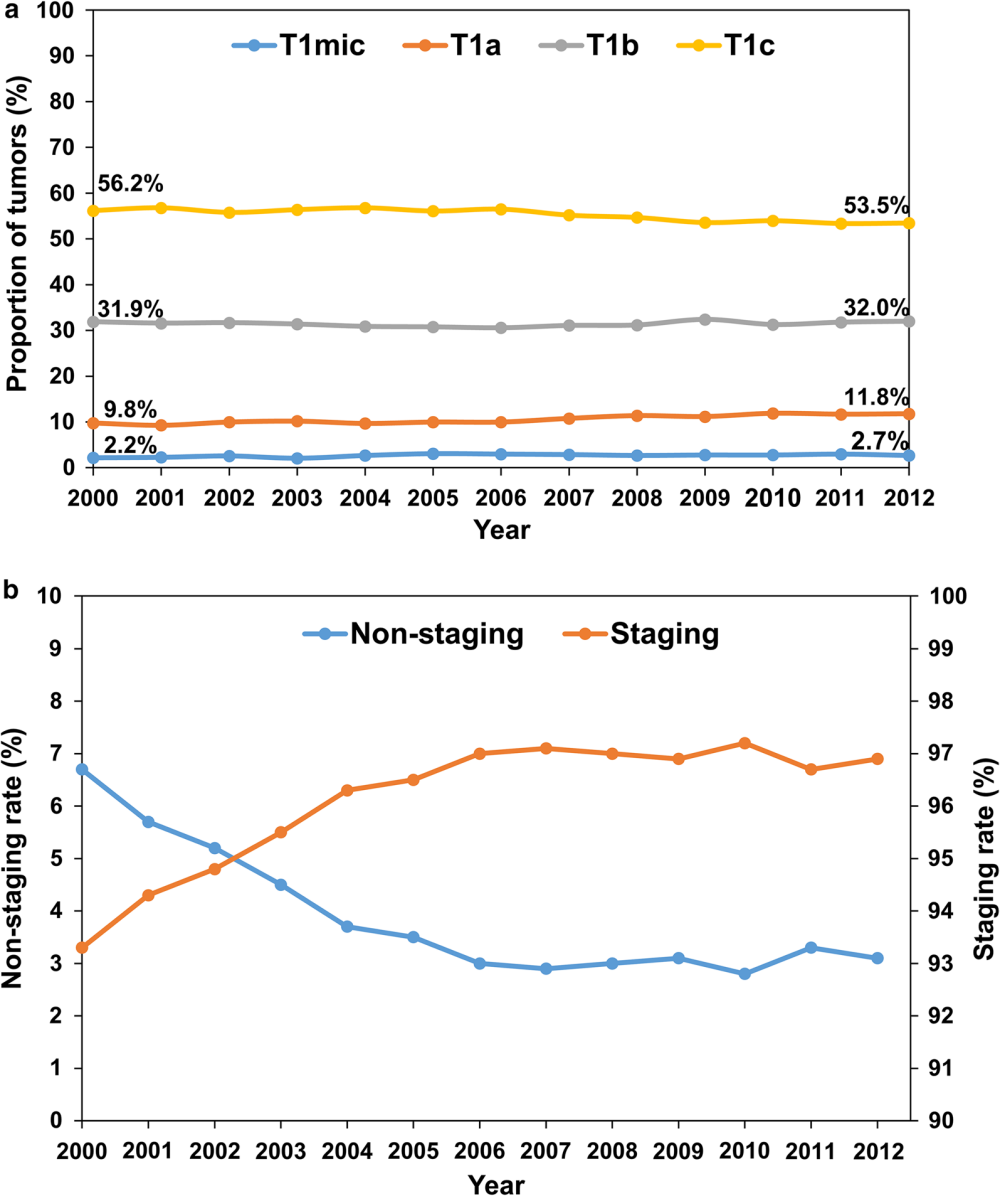
Temporal trends of tumor size and surgical axillary staging in the 166,615 T1 breast cancer patients. **a** Tumor size distribution; **b** rate of surgical axillary staging

A total of 406,535 women older than 18 years were diagnosed with T1 breast cancer between January 1, 2000 and December 31, 2012. We identified 166,615 eligible patients who were treated with BCS and radiotherapy. Among them, 160,141 (96.1%) patients who underwent SLNB or ALND were classified into the staging group, and 6474 (3.9%) patients who had no lymph node examination or only needle aspiration biopsy of lymph nodes were classified into the non-staging group (Fig. 1). The proportions of T1mic and T1a tumors increased with years, followed by a significant decrease in the proportion of T1c tumors from 56.2% to 53.5% (*P* < 0.001) (Fig. 2a). Although the tumor size at presentation was decreasing over years, the rate of surgical axillary staging increased from 93.3% to 96.9% (*P *< 0.001) (Fig. 2b).

Table 1 summarizes the association of surgical axillary staging with other variables. The median ages of patients in the staging and non-staging groups were 60 and 72 years. In the whole cohort, there were more non-Hispanic white, widowed, and older (> 65 years) patients as well as higher proportions of small (T1mic/T1a), well-differentiated (Grade I), and favorable histological types of tumors in the non-staging group (*P* < 0.001). Additionally, patients in the non-staging group were less likely to receive chemotherapy (*P* < 0.001). Balance in patient characteristics was achieved after propensity score matching (*P* > 0.05). Sensitivity analysis showed a Γ value of 1.253, suggesting that the majority of relevant covariates were included with no significant hidden confounder potentially affecting the treatment effects and that all observed covariates had the same chance of assignment to treatment in the two groups in the matched cohort.

**Table 1:** Characteristics of patients with or without surgical axillary staging

Characteristic	Whole cohort [cases (%)]			Matched cohort [cases (%)]		
	Non-staging group	Staging group	*P*	Non-staging group	Staging group	*P*
Total	6474	160,141		5561	5561	
Diagnosis year			< 0.001			0.871
2000–2003	2779 (42.9)	47,508 (29.7)		2362 (42.5)	2364 (42.5)	
2004–2008	2054 (31.7)	61,515 (38.4)		1759 (31.6)	1737 (31.2)	
2009–2012	1641 (25.3)	51,118 (31.9)		1440 (25.9)	1460 (26.3)	
Race			< 0.001			0.423
NHW	5130 (79.2)	124,187 (77.5)		4669 (84.0)	4601 (82.7)	
NHB	528 (8.2)	11,880 (7.4)		353 (6.3)	353 (6.3)	
NHAIAN	22 (0.3)	681 (0.4)		7 (0.1)	9 (0.2)	
NHAPI	342 (5.3)	10,660 (6.7)		227 (4.1)	260 (4.7)	
Hispanic	430 (6.6)	12,286 (7.7)		301 (5.4)	335 (6.0)	
Unknown	22 (0.3)	447 (0.3)		4 (0.1)	3 (0.1)	
Marital status			< 0.001			0.236
Married	3089 (47.7)	99,060 (61.9)		2823 (50.8)	2738 (49.2)	
Never married	639 (9.9)	17,685 (11.0)		474 (8.5)	491 (8.8)	
Widowed	1856 (28.7)	19,817 (12.4)		1617 (29.1)	1612 (29.0)	
Divorced	630 (9.7)	18,539 (11.6)		480 (8.6)	531 (9.5)	
Unknown	260 (4.0)	5040 (3.1)		167 (3.0)	189 (3.4)	
Age (years)			< 0.001			0.525
18–49	530 (8.2)	31,434 (19.6)		435 (7.8)	478 (8.6)	
50–64	1623 (25.1)	69,378 (43.3)		1432 (25.8)	1394 (25.1)	
65–79	2596 (40.1)	51,559 (32.2)		2299 (41.3)	2285 (41.1)	
80–	1725 (26.6)	7770 (4.9)		1395 (25.1)	1404 (25.2)	
T stage			<0.001			0.905
T1mic	667 (10.3)	3778 (2.4)		423 (7.6)	420 (7.6)	
T1a	1328 (20.5)	16,354 (10.2)		1105 (19.9)	1121 (20.2)	
T1b	2020 (31.2)	50,343 (31.4)		1804 (32.4)	1827 (32.9)	
T1c	2459 (38.0)	89,666 (56.0)		2229 (40.1)	2193 (39.4)	
N stage			NA			NA
N0	NA	134,137 (83.8)		NA	4977 (89.5)	
N1	NA	22,617 (14.1)		NA	527 (9.5)	
N2	NA	2552 (1.6)		NA	44 (0.8)	
N3	NA	835 (0.5)		NA	13 (0.2)	
Histological type			<0.001			0.330
Ductal	4616 (71.3)	122,938 (76.8)		4149 (74.6)	4079 (73.4)	
Lobular	1030 (15.9)	26,723 (16.7)		850 (15.3)	919 (16.5)	
Favorable	676 (10.4)	8284 (5.2)		504 (9.1)	501 (9.0)	
Others	152 (2.3)	2196 (1.4)		58 (1.0)	62 (1.1)	
Grade			< 0.001			0.911
I	2180 (33.7)	48,713 (30.4)		1934 (34.8)	1965 (35.3)	
II	2505 (38.7)	68,905 (43.0)		2270 (40.8)	2263 (40.7)	
III	1048 (16.2)	35,381 (22.1)		863 (15.5)	853 (15.4)	
Unknown	741 (11.4)	7142 (4.5)		494 (8.9)	480 (8.6)	
ER			< 0.001			0.776
Negative	678 (10.5)	20,623 (12.9)		518 (9.3)	540 (9.7)	
Positive	5006 (77.3)	132,246 (82.6)		4511 (81.1)	4492 (80.8)	
Unknown	790 (12.2)	7272 (4.5)		532 (9.6)	529 (9.5)	
PR			< 0.001			0.861
Negative	1357 (21.0)	36,191 (22.6)		1087 (19.5)	1107 (19.9)	
Positive	4211 (65.0)	114,492 (71.5)		3881 (69.8)	3855 (69.3)	
Unknown	906 (14.0)	9458 (5.9)		593 (10.7)	599 (10.8)	
Chemotherapy			< 0.001			0.246
No	5947 (91.9)	112,270 (70.1)		5109 (91.9)	5075 (91.3)	
Yes	527 (8.1)	47,871 (29.9)		452 (8.1)	486 (8.7)	

*NHW* non-Hispanic white, *NHB* non-Hispanic black, *NHAIAN* non-Hispanic American Indian/Alaska native, *NHAPI* non-Hispanic Asian or Pacific Islander, *NA* not available, *ER* estrogen receptor, *PR* progesterone receptor

### Multivariate analysis of BCSS

**Fig. 3:**
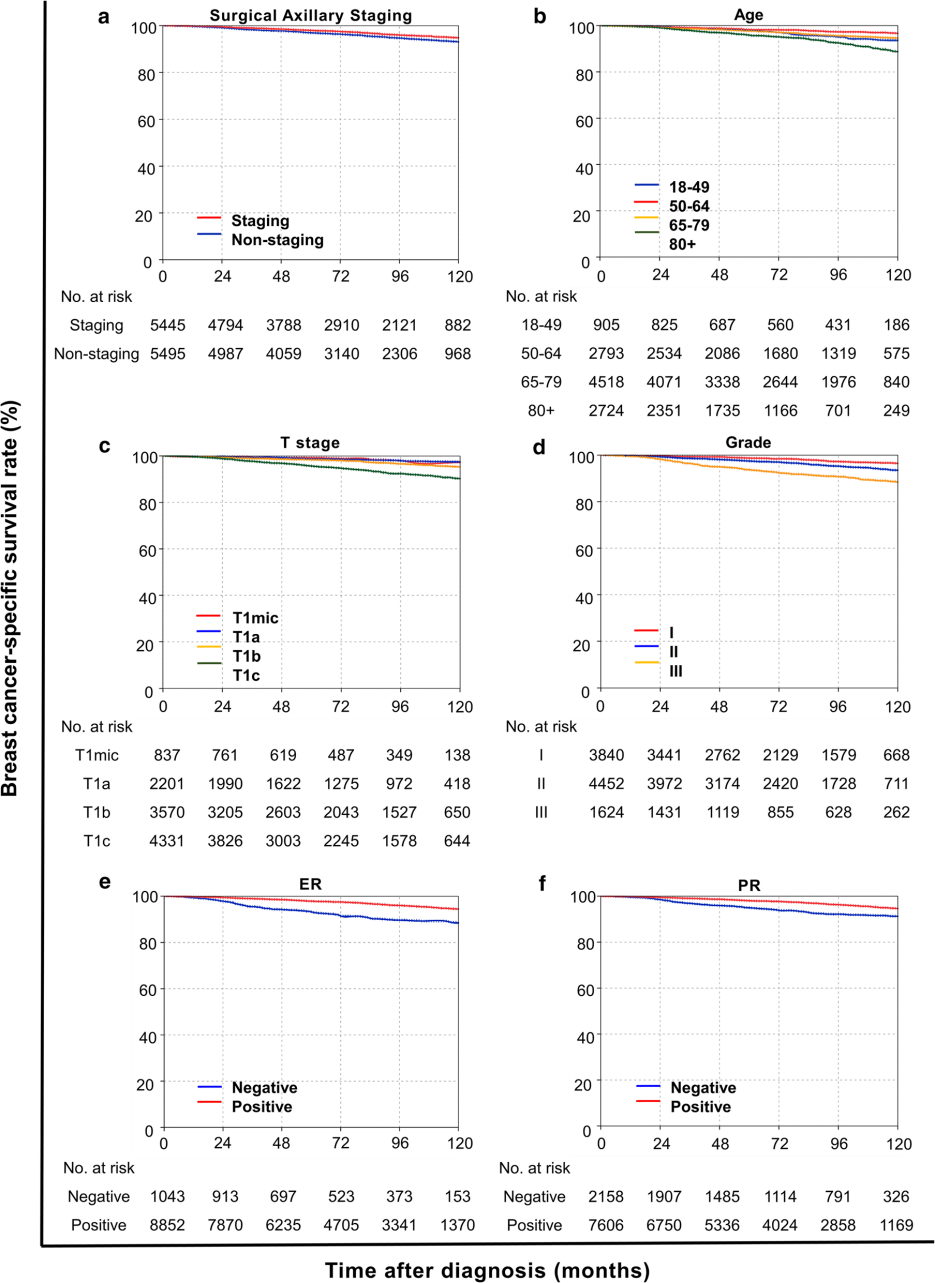
Kaplan–Meier curves of breast cancer-specific survival (BCSS) in the matched cohort. **a** Surgical axillary staging significantly prolonged the BCSS of patents (*P* < 0.001). Patients with age between 50 and 64 years old (**b**), T1mic/T1a tumor (**c**), grade I disease (**d**), positive estrogen receptor (ER) (**e**), and positive progesterone receptor (PR) (**f**) had longer BCSS than their counterparts (all *P* < 0.05)

Median follow-up of the matched cohort was 89 months (interquartile range 52–134 months), which was the same as that of the whole cohort. The 5-year BCSS rates of the whole and matched cohorts were 98.0% and 97.5%. As shown in Table 2, BCSS was improved over time, and all the variables were identified to be significantly associated with BCSS in the whole cohort. For the matched cohort, the risk of death from breast cancer in patients with surgical axillary staging was significantly lower than in the non-staging group (HR = 0.70, 95% CI 0.59–0.83, *P* < 0.001) (Fig. 3a). Patients with an age between 50 and 64 years, T1mic/T1a tumor, grade I disease, positive estrogen receptor (ER) status, and positive progesterone receptor (PR) status had longer BCSS than their counterparts (Fig. 3b–f). The use of chemotherapy did not show a survival benefit in multivariate analysis (HR = 1.29, 95% CI 0.94–1.77, *P* = 0.115), nor did favorable histological types (HR = 0.99, 95% CI 0.69–1.42, *P* = 0.966) (Table 2). In addition, American Indian/Alaska native (HR = 4.73, 95% CI 1.16–19.27, *P* = 0.030) and widowed patients (HR = 1.25, 95% CI 1.01–1.55, *P* = 0.045) had shorter BCSS relative to other groups (Table 2).

**Table 2:** Multivariate analysis of BCSS in the whole and matched cohorts

Variable	Whole cohort		Matched cohort	
	HR (95% CI)	*P*	HR (95% CI)	*P*
Diagnosis year				
2000–2003	Ref.		Ref.	
2004–2008	0.76 (0.72–0.80)	< 0.001	0.69 (0.56–0.86)	0.001
2009–2012	0.63 (0.57–0.69)	< 0.001	0.64 (0.45–0.92)	0.014
Race				
NHW	Ref.		Ref.	
NHB	1.51 (1.39–1.64)	< 0.001	1.27 (0.91–1.78)	0.157
NHAIAN	1.42 (1.01–1.99)	0.047	4.73 (1.16–19.27)	0.030
NHAPI	0.89 (0.79–0.99)	0.047	1.29 (0.83–2.00)	0.266
Hispanic	1.11 (1.01–1.22)	0.036	0.94 (0.61–1.44)	0.760
Unknown	0.45 (0.20–1.01)	0.050	0.00 (0.00–12.40)	0.925
Marital status				
Married	Ref.		Ref.	
Single	1.17 (1.08–1.27)	< 0.001	1.10 (0.78–1.57)	0.571
Widowed	1.26 (1.17–1.36)	< 0.001	1.25 (1.01–1.55)	0.045
Divorced	1.23 (1.13–1.33)	< 0.001	1.15 (0.82–1.62)	0.416
Unknown	1.14 (0.98–1.32)	0.095	0.97 (0.54–1.74)	0.914
Age (years)				
18–49	Ref.		Ref.	
50–64	0.96 (0.89–1.03)	0.200	0.69 (0.48–0.99)	0.042
65–79	1.69 (1.56–1.83)	< 0.001	1.13 (0.81–1.57)	0.466
80–	3.08 (2.74–3.46)	< 0.001	1.72 (1.23–2.41)	0.001
T stage				
T1mic	Ref.		Ref.	
T1a	1.29 (0.76–1.68)	0.065	1.35 (0.76–2.40)	0.309
T1b	1.78 (1.14–2.28)	< 0.001	1.94 (1.14–3.33)	0.015
T1c	2.93 (2.15–3.73)	< 0.001	3.62 (2.15–6.10)	< 0.001
Histological type				
Ductal	Ref.		Ref.	
Lobular	0.94 (0.87–1.01)	0.077	0.87 (0.67–1.13)	0.295
Favorable	0.61 (0.52–0.72)	< 0.001	0.99 (0.69–1.42)	0.966
Others	0.69 (0.57–0.83)	< 0.001	0.93 (0.46–1.88)	0.840
Grade				
I	Ref.		Ref.	
II	1.75 (1.62–1.90)	< 0.001	1.62 (1.27–2.06)	< 0.001
III	2.68 (2.45–2.93)	< 0.001	2.50 (1.89–3.32)	< 0.001
Unknown	1.81 (1.58–2.09)	< 0.001	1.32 (0.88–2.00)	0.180
ER				
Negative	Ref.		Ref.	
Positive	0.73 (0.67–0.80)	< 0.001	0.67 (0.48–0.92)	0.013
Unknown	0.69 (0.57–0.85)	< 0.001	0.38 (0.19–0.75)	0.006
PR				
Negative	Ref.		Ref.	
Positive	0.74 (0.69–0.79)	< 0.001	0.74 (0.57–0.97)	0.027
Unknown	1.00 (0.83–1.20)	0.998	1.48 (0.80–2.72)	0.213
Chemotherapy				
No	Ref.		Ref.	
Yes	1.49 (1.39–1.58)	< 0.001	1.29 (0.94–1.77)	0.115
Surgical axillary staging				
No	Ref.		Ref.	
Yes	0.68 (0.60–0.76)	< 0.001	0.70 (0.59–0.83)	< 0.001

*BCSS* breast cancer-specific survival, *HR* hazard ratios, *CI* confidence intervals, *NHW* non-Hispanic white, *NHB* non-Hispanic black, *NHAIAN* non-Hispanic American Indian/Alaska native, *NHAPI* non-Hispanic Asian or Pacific Islander, *ER* estrogen receptor, *PR* progesterone receptor

### Stratified analysis of BCSS within the matched cohort

As shown in Table 3, surgical axillary staging significantly prolonged BCSS of patients younger than 50 years (HR = 0.45, 95% CI 0.24–0.86, *P* = 0.015) or not younger than 80 years (HR = 0.64, 95% CI 0.47–0.86, *P* = 0.004) (Additional file 1: Figure S1). Patients with smaller tumors (< 1 cm) had similar BCSS between the two groups (*P* > 0.05). However, for patients with T1c tumors, BCSS was significantly longer in the staging group than in the non-staging group (HR = 0.67, 95% CI 0.54–0.84, *P* = 0.001) (Additional file 2: Figure S2). The exemption of surgical axillary staging was safe in patients with grade I disease (HR = 0.98, 95% CI 0.66–1.46, *P* = 0.933) (Additional file 3: Figure S3) or favorable histological types (HR = 0.91, 95% CI 0.47–1.75, *P* = 0.777) (Additional file 4: Figure S4). Race, marital status, hormone receptors, and chemotherapy were not associated with the favorable effect of surgical axillary staging on BCSS in the stratified analysis (all *P* > 0.05) (data not shown).

**Table 3:** Stratified analysis of BCSS in the matched cohort

Variable	Surgical axillary staging	Univariate		Multivariate	
		HR (95% CI)	*P*	HR (95% CI)	*P*
Age (years)					
18–49	No	Ref.		Ref.	
	Yes	0.49 (0.27–0.90)	0.021	0.45 (0.24–0.86)	0.015
50–64	No	Ref.		Ref.	
	Yes	0.79 (0.51–1.22)	0.289	0.82 (0.53–1.27)	0.374
65–79	No	Ref.		Ref.	
	Yes	0.80 (0.61–1.06)	0.116	0.78 (0.59–1.02)	0.070
80–	No	Ref.		Ref.	
	Yes	0.67 (0.50–0.91)	0.010	0.64 (0.47–0.86)	0.004
T stage					
T1mic	No	Ref.		Ref.	
	Yes	1.01 (0.38–2.68)	0.989	0.94 (0.35–2.53)	0.906
T1a	No	Ref.		Ref.	
	Yes	0.74 (0.42–1.30)	0.290	0.73 (0.42–1.30)	0.288
T1b	No	Ref.		Ref.	
	Yes	0.74 (0.52–1.05)	0.090	0.72 (0.51–1.03)	0.069
T1c	No	Ref.		Ref.	
	Yes	0.70 (0.56–0.88)	0.002	0.67 (0.54–0.84)	0.001
Grade					
I	No	Ref.		Ref.	
	Yes	0.98 (0.66–1.45)	0.918	0.98 (0.66–1.46)	0.933
II	No	Ref.		Ref.	
	Yes	0.77 (0.59–1.01)	0.056	0.74 (0.57–0.97)	0.029
III	No	Ref.		Ref.	
	Yes	0.51 (0.36–0.71)	< 0.001	0.49 (0.35–0.68)	< 0.001
Unknown	No	Ref.		Ref.	
	Yes	0.98 (0.52–1.83)	0.936	0.92 (0.49–1.72)	0.791
Histological type					
Ductal	No	Ref.		Ref.	
	Yes	0.75 (0.62–0.92)	0.005	0.72 (0.59–0.88)	0.001
Lobular	No	Ref.		Ref.	
	Yes	0.62 (0.38–0.97)	0.045	0.63 (0.39–0.99)	0.048
Favorable	No	Ref.		Ref.	
	Yes	0.96 (0.50–1.84)	0.896	0.91 (0.47–1.75)	0.777
Others	No	Ref.		Ref.	
	Yes	0.28 (0.06–1.38)	0.118	0.29 (0.06–1.41)	0.124

*BCSS* breast cancer-specific survival, *HR* hazard ratios, *CI* confidence intervals

## Discussion

The risk of lymph node metastasis in patients with ductal carcinoma in situ (DCIS) is estimated to be only 1%–6%, for whom surgical axillary staging is not required according to the National Comprehensive Cancer Network (NCCN) guidelines [23, 24]. In the present study, the rates of lymph node metastasis in patients with T1mic, T1a, T1b, and T1c tumors were 2.8%, 4.5%, 9.3% and 21.0%, respectively. Therefore, it seems reasonable to omit surgical axillary staging for patients with T1mic or T1a tumors. Furthermore, our survival analysis showed no difference in BCSS between staging and non-staging groups in patients with T1mic, T1a, and T1b tumors, whereas surgical axillary staging only prolonged BCSS of patients with T1c breast cancer.

Young breast cancer patients often present with a more advanced stage and aggressive subtypes at diagnosis, resulting in a poorer prognosis [2]. However, there is a paucity of data regarding the safety of treating young women with less aggressive axillary surgery. A randomized trial (INT09/98) was conducted to determine the impact of avoiding axillary surgery in patients with T1N0 breast cancer [25]. In that trial, 517 patients aged 30–65 years with T1N0 breast cancer were recruited between 1998 and 2003 and were randomized to undergo quadrantectomy either with or without ALND. After a median follow-up of 10 years, no difference was observed in overall survival (OS) and disease-free survival (DFS) between the two treatment arms [25]. In the current study, BCSS did not differ between the two treatment arms in patients aged 50–65 years, which is partially consistent with the results of the INT09/98 trial. However, we identified that surgical axillary staging significantly prolonged BCSS in patients younger than 50 years, suggesting that we should still adhere to the current standard treatment for premenopausal patients.

Breast cancer patients older than 65 years tend to have a favorable prognosis and may not benefit from surgical treatment of the axillary lymph nodes [26, 27]. A study began in 1996 recruited 65–80-year-old patients with cT1N0 breast cancer who were randomized to undergo conservative surgery with or without ALND. After 15 years of follow-up, breast cancer-specific mortality and OS did not differ between the ALND and no ALND arms, and the rates of distant metastases were also indistinguishable [28]. However, Sun et al. [26] found that forgoing surgical axillary treatments in women older than 65 years was associated with short OS and BCSS. The controversy among these trials might be attributed to the relatively small sample sizes. Our results showed that it was safe to omit surgical axillary staging in women between 50 and 79 years old. However, we also found that surgical axillary staging significantly prolonged BCSS of patients of at least 80 years old. We speculate that patients of at least 80 years old were less likely to receive standard systemic therapy than younger patients.

Marrazzo et al. [29] indicated that patients with triple-negative breast cancer could be good candidates for BCT without surgical axillary staging. However, in the present study, ER/PR status was not significantly associated with the impact of surgical axillary staging on BCSS. Although adjuvant chemotherapy has been shown to reduce 10-year breast cancer mortality for all subtypes by one-third compared with no chemotherapy [30], patients who were at low risk for recurrence had a small absolute benefit which might be outweighed by long-term toxicities [31]. Consistently, our results showed that chemotherapy did not prolong BCSS of patients with T1 breast cancer either.

Since the importance of surgical axillary staging is still debatable, new ongoing trials, including the Sentinel Node versus Observation after Axillary Ultrasound (SOUND) trial [32] and the Intergroup Sentinel Mamma (INSEMA) trial [33], have been designed to compare SLNB versus observation in cT1-2N0 patients treated with BCT. The SOUND trial, a non-inferiority trial, aimed to recruit 1560 women (780 in each arm), with the primary endpoint being DFS and OS. The INSEMA study planned to randomize patients to either no axillary surgical intervention or SLNB in a 1:4 allocation (1348 patients in no intervention arm). In the present study, each treatment arm in the matched cohort included 5561 patients, and all covariates were comparable after propensity score matching, which was identified as a simulation of randomized clinical trials [20].

Owing to its retrospective nature, the present study had several limitations. Because the SEER database does not have a reliable parameter to distinguish between ALND and SLNB, we assumed that lymph node examination number ≥ 1 represented a formal surgical axillary staging. In general, radiotherapy after BCS is supposed to cover the whole breast with or without regional nodes, which may influence axillary recurrence rates in patients with low-volume axillary disease [6]. In the present study, all patients underwent BCT. However, information regarding the extent and dose of irradiation was not available. Additionally, endocrine therapy was not recorded, but we believe that this might have not largely impacted the results of this study because the majority of patients with early-stage breast cancer who completed appropriate locoregional treatment were likely to undergo standard systemic therapy [34].

In terms of strong preconceptions on the potential therapeutic benefit of axillary surgery, many patients and physicians are unwilling to take the risk for choosing less aggressive surgical management of the axilla, thereby making randomization problematic. Therefore, a large retrospective study might be an ideal design alternative to solve this dilemma [35]. Due to the great disparity in the proportion of patients with or without surgical axillary staging, it is difficult to avoid selection bias. The multivariate model using Cox regression analysis alone may not fully adjust many confounding factors. Therefore, we performed greedy matching techniques to balance all measured covariates in the dataset, which is a pseudo-randomized study design. Further, we used propensity score matching, which is a widely accepted approach for the control of selection bias in observational studies [36].

## Conclusions

Due to more effective screening strategies and adjuvant therapies, the potential risks of axillary surgery may now outweigh its potential benefits, especially in early-stage breast cancer patients treated with BCT. Before the results of ongoing clinical trials are announced, findings of the present mono-institutional retrospective study hint a rationale for waiving surgical axillary staging in subgroups of T1 breast cancers, which are characterized as having tumor size < 1 cm, being 50–79 years old, having grade I disease, and favorable histological types. The possibility to de-escalate axillary treatments needs to be further investigated according to the molecular features of the primary tumor, to be more cost-effective and to reduce risks of potentially avoidable morbidity.

## Additional files

**Additional file 1: Figure S1.** Breast cancer-specific survival (BCSS) curves of patients with pT1 breast carcinoma in the staging and non-staging groups stratified by age.

**Additional file 2: Figure S2.** Effect of surgical axillary staging on BCSS stratified by T stage.

**Additional file 3: Figure S3.** Effect of surgical axillary staging on BCSS stratified by grade.

**Additional file 4: Figure S4.** Effect of surgical axillary staging on BCSS stratified by histological type.

## Abbreviations

BCT: breast-conserving therapy
SEER: Surveillance, Epidemiology, and End Results
SLNB: sentinel lymph node biopsy
ALND: axillary lymph node dissection
BCSS: breast cancer-specific survival
PSM: propensity score matching
HR: hazard ratios
CI: confidence interval
ER: estrogen receptor
PR: progesterone receptor
