# Supplementary figures and images for: Is surgical axillary staging necessary in women with T1 breast cancer who are treated with breast-conserving therapy?
Source: Cancer Commun (Lond). 2019 May 8;39:25. doi: 10.1186/s40880-019-0371-y (PMC6505128; doi:10.1186/s40880-019-0371-y)

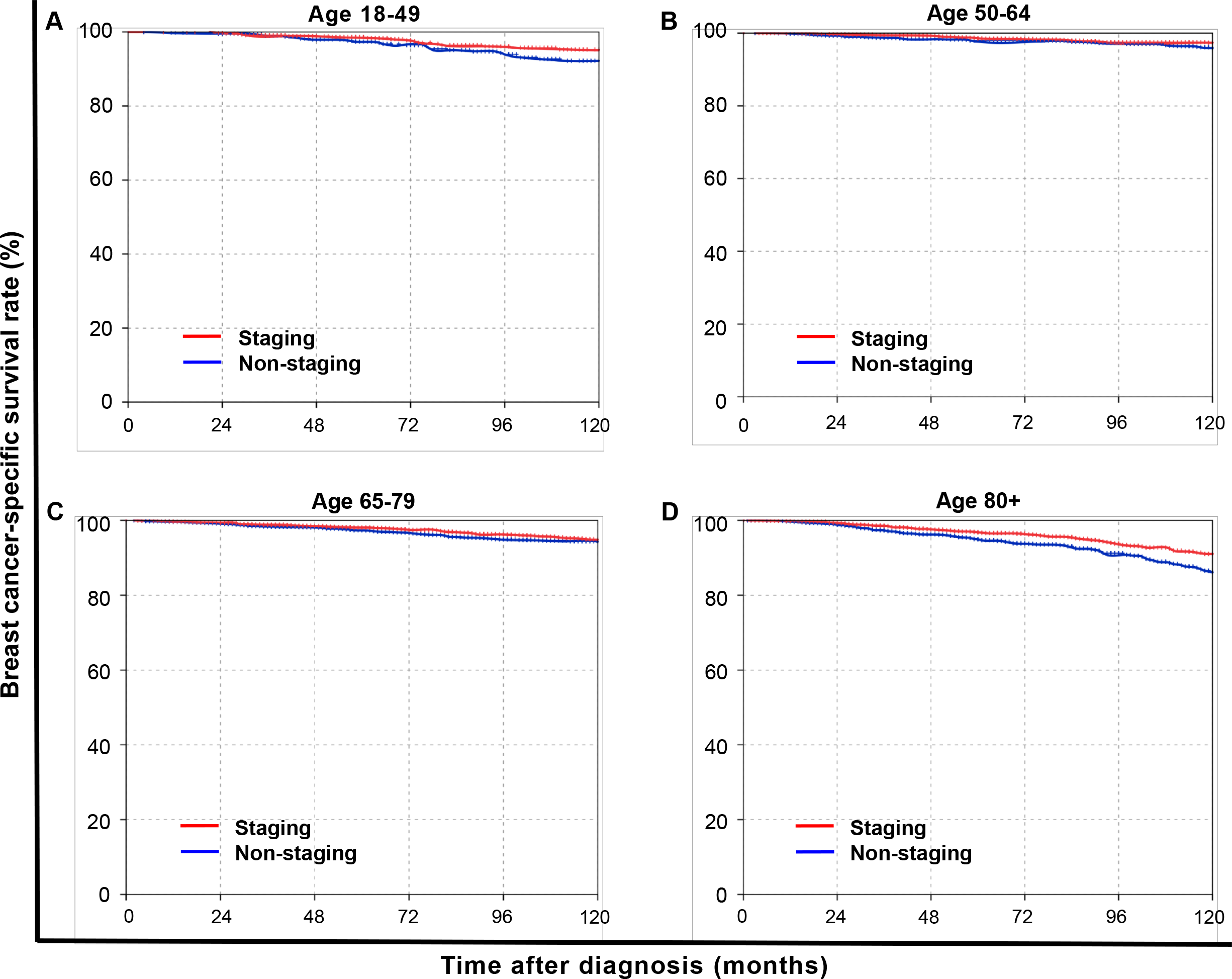

Supplement: Supplementary file 1 — Additional file 1: Figure S1. Breast cancer-specific survival (BCSS) curves of patients with pT1 breast carcinoma in the staging and non-staging groups stratified by age. [file 40880_2019_371_MOESM1_ESM.tif]

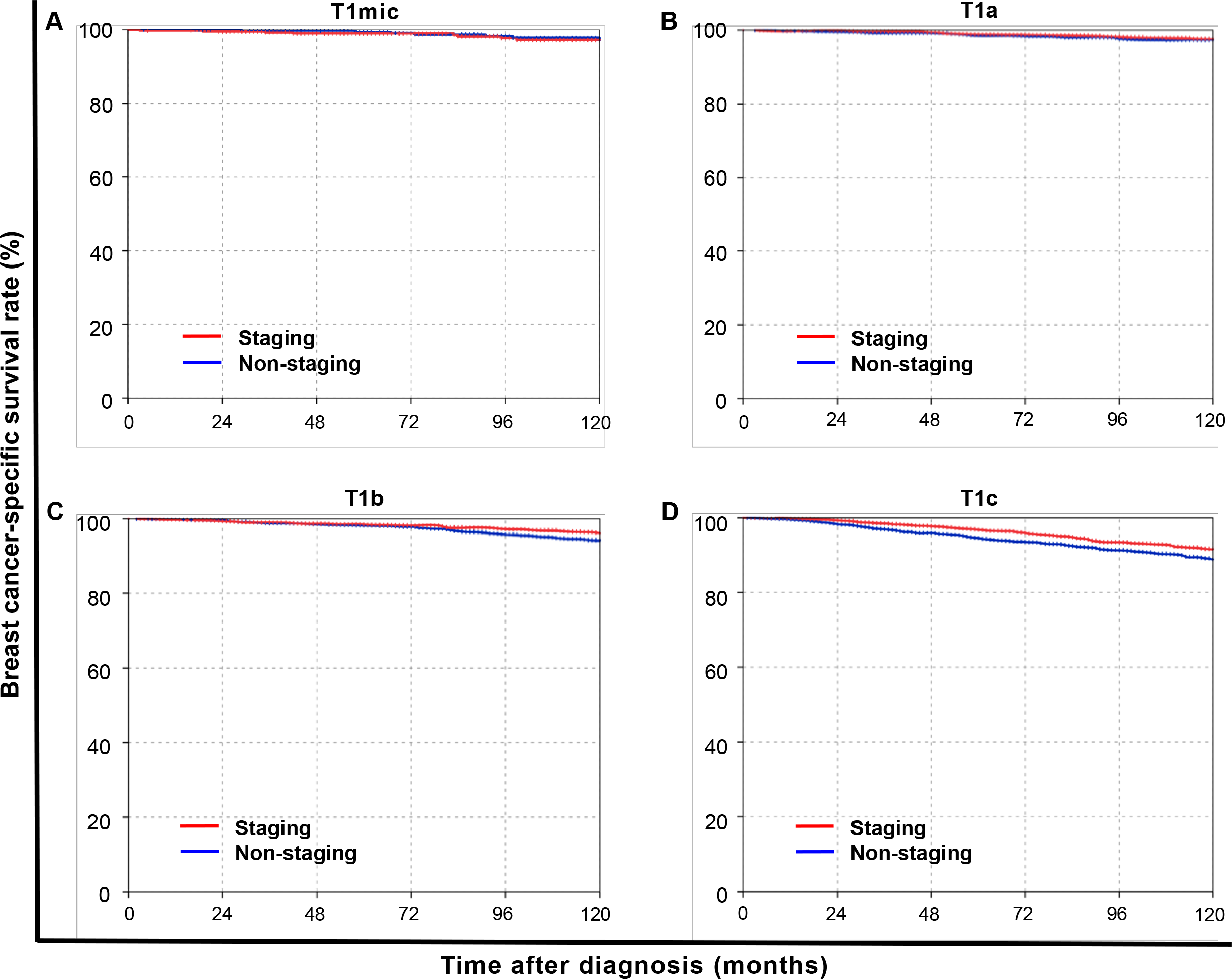

Supplement: Supplementary file 2 — Additional file 2: Figure S2. Effect of surgical axillary staging on BCSS stratified by T stage. [file 40880_2019_371_MOESM2_ESM.tif]

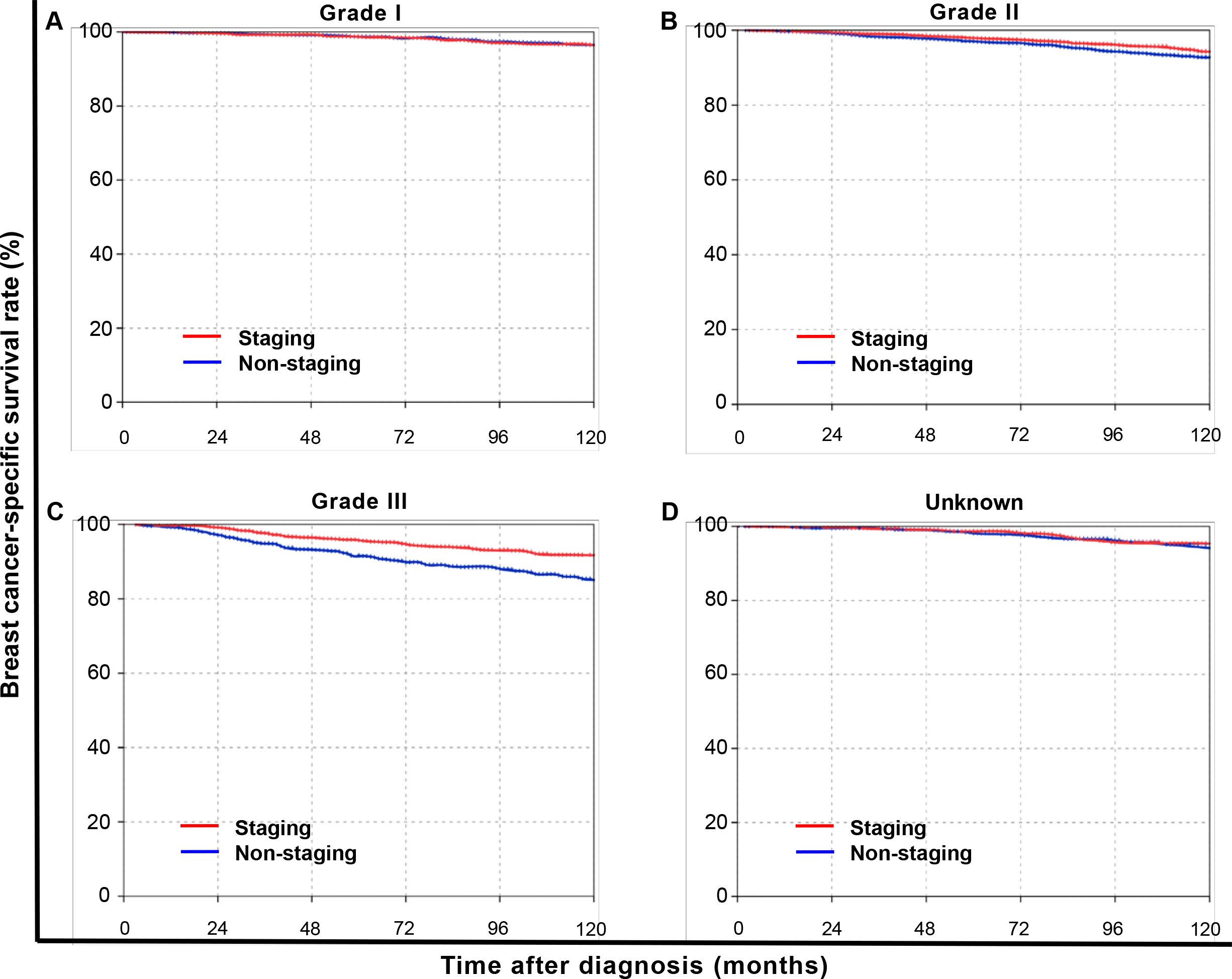

Supplement: Supplementary file 3 — Additional file 3: Figure S3. Effect of surgical axillary staging on BCSS stratified by grade. [file 40880_2019_371_MOESM3_ESM.tif]

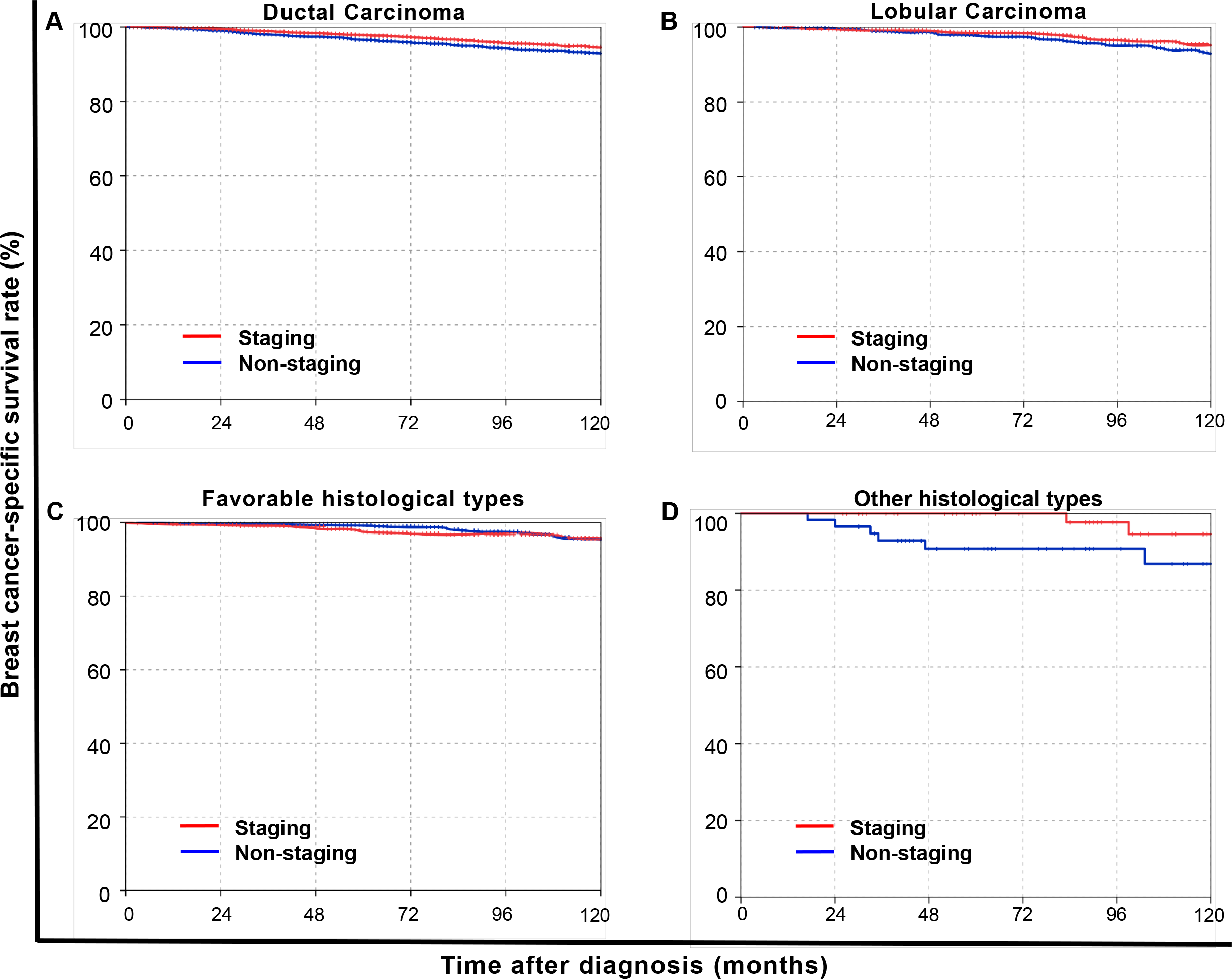

Supplement: Supplementary file 4 — Additional file 4: Figure S4. Effect of surgical axillary staging on BCSS stratified by histological type. [file 40880_2019_371_MOESM4_ESM.tif]
